# Two cases of COVID-19 presenting with severe malaria: a clinical challenge (case report)

**DOI:** 10.11604/pamj.2023.44.83.34453

**Published:** 2023-02-09

**Authors:** Nana Kwame Ayisi-Boateng, Kwadwo Boampong, Betty Nkansah Osei Mensah, Eric Oduro, Kingsley Badu

**Affiliations:** 1 Department of Medicine, Kwame Nkrumah University of Science and Technology, Kumasi, Ghana,; 2 University Hospital, Kwame Nkrumah University of Science and Technology, Kumasi, Ghana,; 3 Department of Theoretical and Applied Biology, Kwame Nkrumah University of Science and Technology, Kumasi, Ghana

**Keywords:** Malaria, COVID-19, sub-Saharan Africa, Ghana, case report

## Abstract

The novel coronavirus (COVID-19) pandemic has stretched the medical resources of both developed and developing countries. The global focus on COVID-19 may lead to the neglect of other infectious diseases such as malaria which is still endemic in many African countries. Some similarities in malaria and COVID-19 disease presentations may also lead to late diagnosis of either disease which could complicate the effects. Here, we present two cases of a 6-year-old child and a 17-year-old female who presented to a primary care facility in Ghana with a clinical and microscopy-confirmed diagnosis of severe malaria complicated by thrombocytopenia. As their symptoms worsened with associated respiratory complications, nasopharyngeal samples were taken for real-time polymerase chain reaction (RT-PCR) and tested positive for severe acute respiratory syndrome coronavirus-2 (SARS-CoV-2). Clinicians, policymakers, and public health practitioners should be alert to the variety of presenting symptoms of COVID-19 and its similarity to malaria to mitigate the risk of mortality from either disease.

## Introduction

Cases of COVID-19-associated deaths are rising in sub-Saharan African countries that are already heavily burdened with malaria [1]. In regions where syndemics might occur, it is essential for malaria-COVID-19 co-infections to be detected early for apt and timely management [2]. Unfortunately, similar disease presentation between malaria and early coronavirus disease may obscure the early diagnosis of either disease.

SARS-CoV-2 predominantly affects the respiratory organs of patients, presenting with an extremely heterogenous range of respiratory symptoms. Symptoms include fever, dyspnea, dry cough, and headache. Other reported symptoms include vomiting, diarrhea, dizziness, and general weakness [3,4]. COVID-19 is diagnosed by taking respiratory tract specimens for molecular analysis using techniques such as real-time reverse transcription-polymerase chain reaction (RT-PCR) assays. Antibody-based techniques for COVID-19 diagnosis are also available [5]. Malaria is caused by the protozoan parasite of the genus *Plasmodium* [6]. Although the rate of malaria infections is declining globally, it remains the number one cause of death in many African countries where 90% of malaria cases occur. According to the World Health Organization (WHO), a total of 229 million cases of malaria were recorded in 2019 with 409,000 deaths [7]. Malaria can present with varied and broad flu-like symptoms. These symptoms include fever, headache, general body pains, vomiting, and hemolytic anaemia. Classically, people with malaria have a paroxysmal occurrences of coldness and sweating [8,9]. Malaria is diagnosed through the microscopic examination of blood films for the identification of *Plasmodium* parasites [6]. Early symptoms presented by COVID-19 patients are oddly similar and generic to symptoms of malaria, which can lead to misdiagnosis or delayed diagnosis of either disease [10]. This could possibly lead to more deaths from COVID-19 and malaria and could jeopardize the progress made in the fight against malaria.

In this report, we present the cases of 6- and 17-year-olds with malaria-COVID-19 coinfection as we seek to elucidate how the two infections mimic each other, diagnostic challenges, and proffer recommendations for clinicians, scientists, and policymakers involved in the global fight against Malaria and COVID-19.

## Patient and observation

### Case 1

**Patient information:** a 6-year-old male presented to the Emergency Department of the University Hospital, Kwame Nkrumah University of Science and Technology (KNUST), Kumasi, Ghana with two days’ history of epigastric pain, chest pains, fever, and vomiting. The mother had administered paracetamol syrup prior to reporting to the hospital, but symptoms did not resolve. He had no known chronic illness and no previous history of hospital admission.

**Clinical results:** on examination, the patient looked restless, pale, and anicteric with a temperature of 36.8°C. Vital signs were unremarkable. The liver was palpable, 2 cm below the right subcostal margin, smooth surface, and mildly tender. The patient had low haemoglobin, elevated white blood cell (WBC), low platelets, elevated liver enzymes, and deranged renal function. Hepatitis B (HBsAg) and C (HCV Ab) were non-reactive (Table 1).

**Table 1:** laboratory observations of patient one

Laboratory investigation	Results		
	Day 1	Day 3 (post-blood transfusion)	Day 5
Haemoglobin	6.5 g/dL	8.3g/dl	7.6g/dl
White blood cell (WBC) count	12.05 x 10^3^/µL	10.20 x 10^3^/µL	10.73 x 10^3^/µL
Platelet count	36 x 10^3^/µL	49 x 10^3^/µL	153 x 10^3^/µL
Malaria parasite count	3,600/µL	Not done	No parasites
Alkaline phosphatase (ALP)	815.7 U/L	328 U/L	Not done
Alanine transaminase (ALT)	99.9 U/L	71.2 U/L	Not done
Aspartate transaminase (AST)	75.3 U/L	55.5 U/L	Not done
Gamma glutaryl Transferase (GGT)	160.4 U/L	117.2 U/L	Not done
Total bilirubin	94.0 µmol/L	20.9 µmol/L	Not done
Direct bilirubin	48.8 µmol/L	14 µmol/L	Not done
Indirect bilirubin	45.20 µmol/L	6.9 µmol/L	Not done
Creatinine	64.17 μmol/L	40.49 µmol/L	Not done
Urea	7.27 mmol/L	2.80 mmol/L	Not done
Glomerular filtration rate	226ml/min/1.72m	385 ml/min/1.72m	Not done

**Diagnostic approach and therapeutic intervention:** on the first day, the patient was given intravenous (IV) artesunate 3.0 mg/kg body weight (55 mg) at 0, 12, and 24 hours, IV paracetamol 275 mg three times daily, and other supportive therapy. He was also transfused 366 mL of whole blood.

On the second day of admission, the patient started experiencing fever (temperature of 40.1°C) with chills and rigors, altered level of consciousness (Blantyre coma score of 4/5), and breathing difficulty (respiratory rate 30 cycles per minute). On chest examination, air entry was adequate bilaterally with vesicular breath sounds. His oxygen saturation was 99% on intranasal oxygen at 6 liters per minute. Due to these clinical features, a nasopharyngeal sample was taken for COVID-19. Post-transfusion laboratory investigations on day 3 showed an improvement in the haemoglobin level as well as the liver enzymes (Table 1). Routine urine analysis showed leucocytes - trace, blood +, and protein +.

On admission day 5, he was started on suspension cefpodoxime 100mg bd for 5 days and other treatments continued. Laboratory results retrieved showed no malaria parasites and full blood count showed approximately 1g/dl drop in haemoglobin level and normalization of the platelets. The patient´s temperature (36.5°C), respiratory rate (18 cycles per minute) and level of consciousness (Blantyre coma scale 5/5) progressively improved and were normal.

**Follow-up:** the patient was discharged from the ward to self-isolate at home, awaiting the RT-PCR results which were reported positive for COVID-19. Clinical results and diagnostic approach are summarized in Table 1.

### Case 2

**Patient information:** the patient was a 17-year-old female who is a known asthmatic and presented to the emergency room with a week´s history of persistent post-prandial epigastric pain associated with vomiting, diarrhea, and fever. She had reported to a peripheral clinic where she was given analgesics without relief.

**Clinical results:** at presentation, the patient was lethargic, had a tinge of jaundice, pallor, and had a mild tenderness in the epigastrium, with no palpable abdominal organ. Her temperature was 37.8°C and her pulse rate was 109 beats per minute. Blood pressure (BP) and oxygen saturation were normal. Laboratory results showed malaria parasites of 600,000/μl, reduced haemoglobin level and low platelets, and normal renal function test (Table 2).

**Table 2:** laboratory results of patient two

Laboratory investigation	Result		
	Day 1	Day 3 (post-blood transfusion)	Day 6
Haemoglobin	9.7 g/dL	Not done	7.3g/dl
White blood cell (WBC) count	12.05 x 10^3^/µL	Not done	14.61 x 10^3^/µL
Platelet count	28 x 10^3^/µL	Not done	117 x 10^3^/µL
Malaria parasite count	600,000/µL	187,245/µL	No parasites
Creatinine	97.65 μmol/L	62.4 μmol/L	Not done
Urea	5.17 mmol/L	6.13 mmol/L	Not done

**Diagnostic approach and therapeutic intervention:** a diagnosis of severe malaria with thrombocytopenia and anaemia was made. The patient was started on IV artesunate 125 mg at 0, 12, 24 hours and IV paracetamol 1g every 8 hours for 24 hours and subsequently continued with artemether/lumefantrine 80/480 twice daily for 3 days. The patient´s temperature, BP, pulse rate, and other vital signs were monitored every 4 hours, as the patient´s temperature continued to rise. Within 12 hours, the highest temperature recorded was 39°C, the BP dropped to 68/38 mmHg and the patient started experiencing respiratory distress. On examination, the patient had a respiratory rate of 35 cycles per minute and flaring of the ala nasi. Chest examination revealed adequate air entry bilaterally, vesicular breath sounds, and bilateral wheezes. Oxygen saturation on room air was 45%. The impression made was severe acute respiratory distress syndrome (ARDS) with a differential diagnosis of septic shock, and acute exacerbation of asthma, to rule out COVID-19. The patient was propped up in bed at 45°C, given oxygen at 10 liters/minute via a non-rebreather mask, and intravenous paracetamol, and was resuscitated with intravenous fluids. A urethral catheter was passed to monitor urine output. She was nebulized with 5 mg of salbutamol for 3 cycles and was given 200 mg of intravenous Hydrocortisone. Oxygen saturation improved to between 75-77% on oxygen, BP 132/78 mmHg, and respiratory rate 40 cpm. The patient was started on intravenous ceftriaxone 2g daily and nebulization with 5 mg of salbutamol was continued.

On the third day of admission, dyspnea had improved, the temperature was 37°C, the pulse rate was 111 bpm, and oxygen saturation of 96% on the non-rebreather mask with the patient propped at an angle of 45°. A chest X-ray taken showed heterogenous opacifications at the upper right lung zones and homogenous opacifications at the lower lung zones. Repeated blood film for malaria parasites showed a reduction in the parasite load (Table 2). The erythrocyte sedimentation rate (ESR) was 100 ml/fall/hr.

On day four of admission, the temperature was 36.7°C, pulse rate 132 bpm, blood pressure of 90/60 mmHg and oxygen saturation was 91%. The impression made was severe malaria complicated by thrombocytopenia, intravascular hemolysis, and acute respiratory distress syndrome with a differential diagnosis of COVID-19. Nasopharyngeal and oropharyngeal samples were taken for COVID-19 testing and the ESR and full blood count were repeated. At this point, the patient was started on tab azithromycin 500 mg immediately and 250 mg daily for 7 days, IV dexamethasone 6 mg daily for 72 hours then tab dexamethasone 6mg daily for 7 days, tab zinc 40 mg daily for 14 days and tab vitamin C 1000 mg daily for 14 days.

On admission day 5, the patient had no new complaints. Vital signs recorded were a temperature of 37.2°C, pulse rate of 83 bpm, blood pressure of 89/62 mmHg, respiratory rate of 32 cpm, and 26 cpm off oxygen and on oxygen, respectively. Oxygen saturation was between 82-84% off oxygen and 96-97% on a non-rebreather mask at 10 L/min. Examination of other systems was unremarkable. All medications were continued.

On day 6, ESR of 100 ml fall/hour. The full blood count showed raised WBC, low haemoglobin level, and higher platelets than on day one (Table 2). The patient´s RT-PCR for COVID-19 was confirmed positive on day 9 of admission. The patient had no complaints and no other symptoms. The patient was jaundiced and warm to touch on examination. Vital signs were a temperature of 36.6°C, pulse rate of 101bpm and blood pressure of 105/75mmHg. Air entry was reduced in the lower lung zones with no added sounds. The rest of the examination was unremarkable. Patient was prescribed tab folic acid 5 mg daily, tab cefuroxime 500 mg every 12 hours, tab artemether/lumefantrine 80/480 mg every 12 hours, tab azithromycin 250 mg daily, syp bioferon 15 ml every 8 hours and tab zinc 30 mg daily.

**Follow-up:** the patient was discharged home on day 17 post-admission. The patient did not report back with any condition.

**Informed consent:** this was obtained from the adult patient and parents of the child. Approval was obtained from the authorities of the University Hospital, KNUST to use data from the patient´s medical records. Privacy, safety, and confidentiality of the patients were strictly ensured.

## Discussion

The effect of the raging COVID-19 outbreak on the incidence of common pathologies and their related deaths in many developing countries is unknown. It is however predictable that diseases such as malaria might be neglected, or essential funds meant to combat such infections would be diverted toward the fight against COVID-19 in these countries. Eventually, preventable deaths from these common pathologies might cause more damage than COVID-19 [11].

The bedrock of clinical success is the timely and accurate diagnosis of diseases. Diseases with overlapping clinical presentations, especially when patients are coinfected, however, present diagnostic and therapeutic challenges to clinicians in primary care delivery. It could delay the diagnosis of the actual disease and cause costly and harmful effects in patients [11,12]. These two cases demonstrate the challenges healthcare practitioners in malaria-endemic regions in the world are facing during the COVID-19 pandemic period.

Malaria patients show symptoms such as fatigue, headache, difficulty in breathing, and fever which are also presented by COVID-19 patients [13-15]. In parts of the world where malaria is the leading cause of hospital admissions, patients showing symptoms such as fever, headache, and general body pains are often suspected of having malaria. Laboratory tests are then run to confirm the presence or absence of plasmodium parasites before patients are administered medications.

Both patients in this report showed symptoms of malaria and this was confirmed through laboratory tests that were run on them. Although both patients were positive for malaria and COVID-19, it is unclear whether the two diseases exacerbated each other´s effect. It was however clear that the patient with high malaria parasite load showed more severe symptoms. The patient in case two had more malaria parasites and exhibited severe symptoms than patient in case one.

Although there are increasing numbers, the spread of COVID-19 has generally been slow in many African countries [16]. Many African countries are however preparing for an exponential rise in the number of COVID-19 cases. This means that more cases of COVID-19-malaria coinfections will be observed in hospitals in these malaria-endemic countries. Many of these countries also lack the diagnostic capacity needed to run many COVID-19 tests. Many people who present with noticeable symptoms such as fever, fatigue, headaches, and difficulty in breathing would be misdiagnosed with either of the diseases in this crisis period if symptoms alone are used to define cases. Even in countries with excellent healthcare systems, about 50% of COVID-19 cases can be missed through symptomatic screening [17]. Although there is a COVID-19 pandemic where it is expected that a high index of suspicion is biased towards COVID-19, it is important that clinicians screen for both diseases if the symptoms presented by patients suggest that they might have either disease. People with fever should not be screened only for COVID-19 and sent home if results are negative. This could lead to the possibility of missing malaria infections and vice versa. Three cases of delayed malaria diagnosis in people presenting with fever have been reported in Israel due to concerns about possible exposure to SARS-CoV-2 [17].

## Conclusion

This report illustrates an exceptionally complicated scenario for the diagnosis of two cases of COVID-19-malaria coinfection. With the rising number of COVID-19 cases in many African countries with high malaria incidence, cases of COVID-19-malaria coinfections should be expected. To prevent the consequence of misdiagnosis or delayed diagnosis, it is recommended that patients who present with infection-suggestive symptoms should be screened thoroughly. Although the COVID-19 pandemic has put a lot of pressure on the healthcare system of many already struggling developing countries, a good balance must be struck in order not to neglect common diseases that are still causing high morbidities and mortalities in these countries.
